# A missense variant in the *PACS2* gene cause Epileptic Encephalopathy and seizures in Saudi family

**DOI:** 10.12669/pjms.40.4.8707

**Published:** 2024

**Authors:** Absarul Haque, Muhammad Imran Naseer

**Affiliations:** 1 Absarul Haque, King Fahd Medical Research Center, Department of Medical Laboratory Technology, Faculty of Applied Medical Sciences, King Abdulaziz University, Jeddah 21589, Saudi Arabia; 2 Muhammad Imran Naseer, Center of Excellence in Genomic Medicine Research, Department of Medical Laboratory Technology, Faculty of Applied Medical Sciences, King Abdulaziz University, Jeddah 21589, Saudi Arabia

**Keywords:** *PACS2* gene, Epileptic encephalopathies, Intellectual disability, Epilepsy, Saudi family

## Abstract

We identified the *PACS2* gene responsible for the multifunctional sorting protein that play a role in nuclear gene expression as well as pathway traffic regulation. Diseases associated with *PACS2* include early infantile epileptic encephalopathy (EIEE66), alacrima, achalasia, and mental retardation syndrome. Whole exome sequencing (WES) technique was used for the identification of variants that may lead to the disease. We identified a consanguineous Saudi family segregating developmental delay, mental retardation and epilepsy. Our results showed a heterozygous missense variant *PACS2* gene leading to intellectual disability, epilepsy and cause epileptic encephalopathies (EIEE66) disorder. WES data was analyzed and identified variants were further confirmed by Sanger sequencing validation technique. We identified a heterozygous missense c.625G>A p.Glu209Lys in exon-6 of *PACS2*. The detected heterozygous mutation in the exon-6 region of *PACS2* gene change the protein features and may cause disease. Further, explain the possibility that *PACS2* gene play important role to cause intellectual disability, epilepsy and epileptic encephalopathies in this Saudi family.

## INTRODUCTION

PACS2 (Phosphofurin Acidic Cluster Sorting Protein 2) is a Protein Coding gene. Endoplasmic reticulum (ER), mitochondria communication along with homeostasis between mitochondria and the ER is controlled by multifunctional sorting protein produced by PASCS2. Further, in reply to translocates BID to mitochondria, apoptotic inducer, that activates series of actions along with the release of cytochrome-C, formation of mitochondrial truncated pro-apoptotic protein BID as well as the activation of caspase-3 leading to the cell death. Moreover, this protein also controls the trafficking of the ion channel, guiding acidic cluster-containing ion channels to different subcellular compartments.^1^ The epileptic encephalopathy may be due to both acquired and genetic causes. This include precise congenital or acquired structural brain abrasions, chromosomal anomalies, metabolic disorders, copy-number variants (CNV), or may be due to the mutation in single gene only.^2^ Mutations in PACS2 can cause epileptic encephalopathy, early infantile, 66 (EIEE66; OMIM 618067) an autosomal dominant disease, with severe infantile and childhood onset epilepsies, characterized by multiple types of seizures, mild to moderate developmental delay, mild hypotonia, septal defect, speech delay, left transverse palmar crease and facial features including down slanting palpebral fissures, thin upper lip, broad nasal root and wide mouth with downturned fatures.^3^,^4^ PACS2 exerts its role both in the nucleus and cytoplasm.

Our patient’s phenotype was consistent with EIEE66, heterozygous missense variant c.625G>A at chr14:g.105834449 NM_001100913.2 in PACS2 predicted to result in substitution of glutamate-to-lysine (p.Glu209Lys) in two individuals with EIEE66 and facial dysmorphism. PACS2 was classified as pathogenic according to the The American College of Medical Genetics and Genomics and the Association for Molecular Pathology (ACMG/AMP) guidelines, and the diagnosis of EIEE66 was confirmed.^5^

## METHODS

**Fig. 1:**
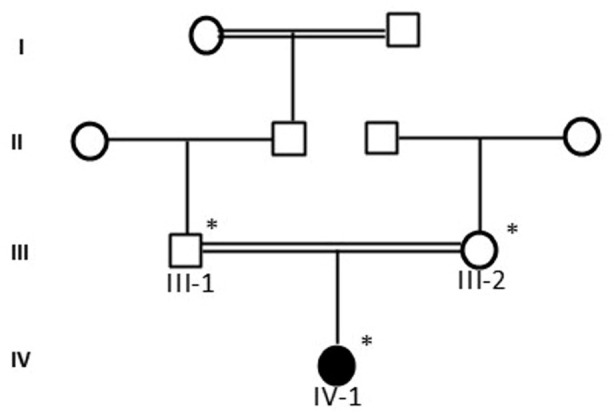
Consanguineous family from Saudi Arabia showing the disease phenotype segregating in an autosomal recessive manner. The sample marked with asterisks was available for genetic testing.

In this study, we took the blood samples from all family members (affected and normal) in line with the institutional ethical guidelines and policies of The King Abdulaziz University Hospital, Jeddah, Saudi Arabia. We also obtained the approval from the family in line with the Helsinki Declaration. This study was done at the Center of Excellence in Genomic Medicine Research, King Abdulaziz University, in 2023 and the study ethical approval number is 013-CEGMR-02-ETH. Non-genetic causes were eliminated. A complete history was taken from the family, after that the pedigree was drawn as shown in Fig-1. DNA was extracted from blood samples and as required for illumina NextSeq instrument with 2x76 paired end reads the sample were prepared.

### Clinical information

The 4-months old male was the only child of consanguineous parents. Family history was unremarkable. The patient was presenting with Focal-onset seizure, Generalized-onset seizure, Neonatal onset seizure. Delivery was at 38 weeks, by Caesarian section because of breech presentation. Weight at birth was 3060 g (25-50th centile), length 50 cm (25th centile), and occipito-frontal circumference (OFC) 36.5 cm (75th centile). At 45^th^ days of life epilepsy were recorded, with seizures characterized by impairment of consciousness and rolling of the eyes. Phenobarbital was started with seizures remission, Brain magnetic resonance imaging (MRI), revealed an abnormal cerebellar foliation pattern along the basal and posterior portions of the right cerebellum hemisphere with reduction of white matter in the supratentorial region.

### Whole exome sequencing (WES)

Extracted DNA from proband was enriched for the coding region and splice site junctions of the genes. We used illumina NextSeq instrument with 2x76 paired end reads. We used hg19, GRCH37/UCSC for reference sequences based on human genome build. Capillary sequencing used to check out the relevant variants with clinical or uncertain significance. All sequence alterations were defined by using the Human Genome Variation society nomenclature guidelines HGMD 2019.4 https://digitalinsights.qiagen.com/news/blog/clinical/hgmd-2019-4-release-2/. By using the gene-specific filtering data were analyzed. We used different bioinformatics tools to identify causative variant in *PACS2* gene.

### Sanger sequencing

The obtained variant after WES analysis were further validated using the Sanger sequencing analysis by designing the targeted primers on the specific area as explained previously.^6^-^8^ BioEdit sequence alignment software was used to align to the corresponding reference sequence.

## RESULTS

**Fig. 2:**
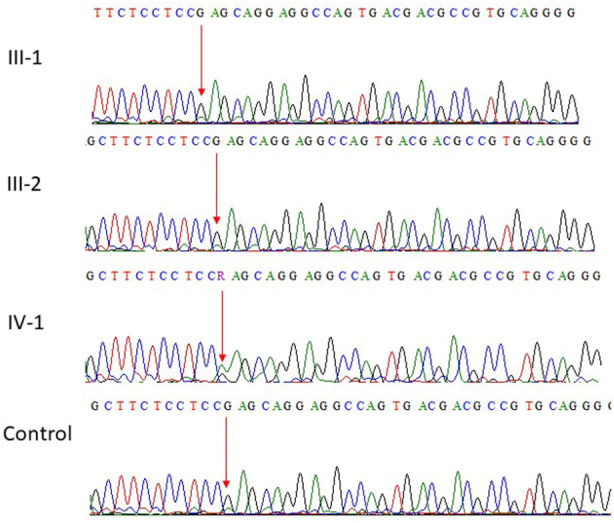
Sanger sequence analysis: **a** and **b** (III-1 and III-2) are normal parents, while (IV-1) is affected child showing G at position 625 replaced by A resultantly amino acid glutamic acid residue into lysine in exon 6 of PACS2 gene.

WES on the sample IV-2 obtained from patient revealed pathogenic variant in *PACS2* gene. The variation is a heterozygous missense c.625G>A p.Glu209Lys in exon 6 where “G” at position 625 replaced by “A” and the resultant amino acid glutamic acid residue into lysine showed a heterozygous missense mutation. The parent’s III-1 and III-2 were wild type homozygous at the same position as shown in Fig.2. The Mutation Tester predicted this disease-causing mutation and was further screened in 100 unrelated healthy persons.

## DISCUSSION

In this study we identified heterozygous missense in *PACS2* c.625G>A p.Glu209Lys exon 6 and this mutation may cause EIEE66. The patient in this study shares common clinical feature, such as seizures, delayed psychomotor development, delayed walking, Intellectual disability (ID), speech delay, focal or multifocal spike abnormalities and facial features.

Recently, 14 unrelated patients with early infantile epileptic encephalopathy-66 (EIEE66; 618067), a de novo heterozygous c.625G-A transition (c.625G-A, NM_001100913.2) in the PACS2 gene, resulting in a glu209-to-lys (E209K) substitution at a highly conserved residue in an acid hydrophobic domain that leads to polarity and protein conformation changes was reported.^5^ Most patients had onset of seizures in the first few days or weeks of life, although one had onset at two months of age. The major seizure types were tonic, focal motor, autonomic, generalized as well as tonic-clonic seizures. EEG showed focal onset or diffuse attenuation with later focal features, with episodes of status epilepticus. Hypsarrhythmia peaks were not observed. The seizures attenuated with time, especially after the first year of life, and a few patients even became seizure free with treatment.^5^ Furthermore, another study identified a *de novo* missense variant, c.625G>A,^4^ and (c.607C>T), in PACS1 (MIM: 607492) in two distinct persons with unexplained ID and strikingly similar facial dysmorphism a heterozygous missense variant.^9^ Recently, three cases having PACS2 gene mutations having dysmorphic facial appearance, development delay and seizure that was cured after the treatment with valproic acid. A recurrent heterozygous missense variant (c.625G>A) in PACS2 gene was reported. Further seventeen patients’ data were also reported in the literature. Moreover, out of twenty cases only two missense variants in PACS2 gene were identified, 19 cases having the c. 625G>A (p.Glu209Lys) variant and one of the case with the variant c. 631G>A (p.Glu211Lys). Early infantile epileptic encephalopathy is related to the PACS2 gene as an autosomal dominant manner disease with dysmorphic facial appearance, seizure onset within the first week of life, and various degrees of developmental abnormalities.^10^

### Limitation of the study

Limitation of this study was availability of blood samples from all the family members who participated in this study.

## CONCLUSIONS

We identified a heterozygous mutation in the *PACS2* gene in our patient with a clear phenotype resembling disease in EIEE66 with facial dysmorphism. The gene variation and the disease presented explain the possibility that *PACS2* gene may play important role and cause intellectual disability, epilepsy and developmental and epileptic encephalopathies in this Saudi family.

### Author Contribution:

**MIN, and AH** designed the experiments.

**MIN and AH** conducted the experiments.

**MIN, AH** analyzed the data and prepared the manuscript.

**AH and MIN** finally revised the manuscript.
